# Comprehensive prediction of immune microenvironment and hot and cold tumor differentiation in cutaneous melanoma based on necroptosis-related lncRNA

**DOI:** 10.1038/s41598-023-34238-0

**Published:** 2023-05-05

**Authors:** Miao Zhang, Lushan Yang, Yizhi Wang, Yuzhi Zuo, Dengdeng Chen, Xing Guo

**Affiliations:** Department of Plastic and Burns Surgery, Affiliated Hospital of Southwest Medical University, Luzhou, Sichuan China

**Keywords:** Computational biology and bioinformatics, Computational models, Functional clustering, Genome informatics, Cancer, Cancer genomics, Cancer microenvironment, Skin cancer, Tumour immunology, Cancer genetics, Cancer genomics, Epigenetics, Immune cell death, Tumour immunology

## Abstract

As per research, causing cancer cells to necroptosis might be used as a therapy to combat cancer drug susceptibility. Long non-coding RNA (lncRNA) modulates the necroptosis process in Skin Cutaneous Melanoma (SKCM), even though the precise mechanism by which it does so has yet been unknown. RNA sequencing and clinical evidence of SKCM patients were accessed from The Cancer Genome Atlas database, and normal skin tissue sequencing data was available from the Genotype-Tissue Expression database. Person correlation analysis, differential screening, and univariate Cox regression were successively utilized to identify necroptosis-related hub lncRNAs. Following this, we adopt the least absolute shrinkage and selection operator regression analysis to construct a risk model. The model was evaluated on various clinical characteristics using many integrated approaches to ensure it generated accurate predictions. Through risk score comparisons and consistent cluster analysis, SKCM patients were sorted either high-risk or low-risk subgroups as well as distinct clusters. Finally, the effect of immune microenvironment, m7G methylation, and viable anti-cancer drugs in risk groups and potential clusters was evaluated in further detail. Included USP30-AS1, LINC01711, LINC00520, NRIR, BASP1-AS1, and LINC02178, the 6 necroptosis-related hub lncRNAs were utilized to construct a novel prediction model with excellent accuracy and sensitivity, which was not influenced by confounding clinical factors. Immune-related, necroptosis, and apoptosis pathways were enhanced in the model structure, as shown by Gene Set Enrichment Analysis findings. TME score, immune factors, immune checkpoint-related genes, m7G methylation-related genes, and anti-cancer drug sensitivity differed significantly between the high-risk and low-risk groups. Cluster 2 was identified as a hot tumor with a better immune response and therapeutic effect. Our study may provide potential biomarkers for predicting prognosis in SKCM and provide personalized clinical therapy for patients based on hot and cold tumor classification.

## Introduction

Melanoma is a type of skin cancer that has a poor prognosis due to its susceptibility to metastasis, its tendency to spread, and its difficulty in detecting in its early stages^1,2^. According to projections released by the International Agency for Research on Cancer, the mortality rate for SKCM patients will rise to 17% in 2020^3^. Even though the pathogenesis is unclear, the potential factors include ultraviolet radiation, melanocytic nevi, dysplastic nevi, genetic susceptibility, and a personal or family history of melanoma^1^. Early and precise diagnosis and targeted treatment planning are essential in preventing tumor progression^4^. With the development of targeted and immunotherapy, patients' overall survival (OS) has improved. However, drug resistance has become more and more common^5^. Therefore, new biomarkers must be explored to improve melanoma's diagnostic and therapeutic accuracy.

Necroptosis is a unique form of programmed cell death that involves a caspase-independent molecular and cellular mechanism, while it is also interconnected to apoptosis and pyroptosis^6^. The activation of receptor-interacting protein kinases 1 (RIPK1) and RIPK3, which in turn triggers alterations in the conformation of mixed lineage kinase domain-like (MLKL) and its translocation to the plasma membrane, is the primary initiating event in necroptosis. Damage-associated molecular patterns (DAMPs) trigger the release of a flood of cytokines, which eventually causes membrane permeabilization to be changed and thereby induce an inflammatory response^7,8^. Necroptosis would have either a pro-tumorigenic or an anti-tumorigenic effect on cancer, relying on the specifics of the tumor's kind, stage, and grade^9,10^. Lack of RIPK1, RIPK3, or MLKL activity is prevalent in some cancers, revealing that induced necroptosis may replace apoptosis as a potential therapy in tumors^11,12^. Researches show that the function of necroptosis in Immuno-Oncology is not unidirectional. The DAMPs-induced inflammation response may promote the progression and metastasis of cancer. On the other hand, it may also promote anti-tumor immunity circumstance according to elevating the cross- stimulation of CD8+ T cells^9,13,14^. Although reactivation of RIPK1 and RIPK3 can lure necroptosis in a melanoma tumor cell to delay tumor progression, the correlation between necroptosis and melanoma still needs further exploration^15,16^.

Long non-coding RNA(LncRNA), low coding or not translating into protein, is a type of transcript with more than 200 nucleotide sequences^17,18^. Most literature thus far suggests that lncRNA aberrant expression and mutations are critical for carcinogenesis, metastasis, and therapeutic resistance in cancer^19,20^. As high-specific tissue and cell drivers of cancer phenotypes, LncRNAs can become a class of potential biomarkers for prognosis and therapy of cancer^21,22^. Moreover, Immunological cell activity and immune response in the tumor microenvironment (TME) can also be altered by lncRNAs, which in turn influencing the efficacy of cancer immunotherapy^23–25^. Several lncRNAs have been associated with melanoma, such as ANRIL, HOTAIR, MALAT1, SAMMSON, etc.^26,27^. However, there is currently no conclusive evidence as to the role of necroptosis-related LncRNAs into melanoma.

This present study attempted to discover some specific necroptosis-associated hub lncRNAs that can predict prognosis, immune environment, and drug sensitivity, alleviating the negative impact of immunotherapy failure in SKCM patients by providing better options. Furthermore, these analyses were also conducted in subgroups, classified as hot and cold tumors, to achieve the purpose of individualized therapy.

## Materials and methods

### Data integration

The RNA sequence datasets were acquired from the TCGA-SKCM cohort and the GTEx-Skin cohort, respectively. Then, we make the data (FPKM values) normalized by the limma R package. Overall samples from both categories added up to 471 tumors and 813 healthy skin samples. Furthermore, the TCGA database was also mined for clinically relevant data on SKCM patients.

### Identification of necroptosis-related lncRNAs

We screened Necroptosis-related genes from the GeneCards database (https://www.genecards.org) according to a correlation score of > 0.5, and searched the Gene Set Enrichment Analysis website (GSEA) (https://www.genecards.org) using the keyword “Necroptosis”. In total, 206 Necroptosis-related genes were collected by the above approach.

Necroptosis-related lncRNAs were detected by Pearson correlation analysis based on coefficients > 0.4 and *P* < 0.001. Next, differential expression necroptosis-related lncRNAs were identified using the limma R package, in which the screening standard is |log2 fold change|> 2 and false discovery rate (FDR) < 0.05.

### Construction of the risk model

Merge clinical information from tumor samples with all differentially expressed lncRNAs data. Overall prognostic impact of necroptosis-associated lncRNAs was evaluated using univariate Cox (uni-Cox) regression analysis to filter potential lncRNAs (*p* < 0.05). Next, a least absolute shrinkage and selection operator (LASSO) regression analysis was run on candidate necroptosis-associated lncRNAs to identify hub lncRNAs and to create a prediction model through multivariate Cox regression. 1000 cycles of LASSO regression analysis were performed using tenfold cross-validation at a significance level of 0.05. Each cycle was randomly grouped 50 times, dividing the sample equally into two groups each time. Furthermore, the LASSO regression results were output only when the error was minimum. The risk score formula is as described below:

$${\text{risk score}} = \mathop \sum \limits_{i = 1}^{n} \exp \left( {lncRNA^{i} } \right)*coef\left( {lncRNA^{i} } \right)$$

where exp(lncRNA) is the relative expression of signature lncRNAs, and coef (lncRNA) is the weighted correlation coefficient of signature lncRNAs. Risk classification of SKCM patients by comparing the risk score of the patient with the median score.

### Constructing and evaluating the nomogram

Univariate cox (uni-Cox) and multiple cox (multi-Cox) regression analyses were performed to assess the relationship between risk score and clinical features. And based on these results, a nomogram was created to predict patient outcomes using the RMS R package. Furthermore, the correction curves were constructed based on the Hosmer–Lemeshow test to verify the predictive reliability regarding the nomogram. The model's 1-, 3- and 5-year time-dependent receiver operating characteristics (ROC) curves were built by the timeROC R package. Finally, we used decision curve analysis (DCA) to further assess the predictive accuracy of this nomogram.

### Gene set enrichment analysis

Determine if there are significant differences throughout the functional pathways between high- and low-risk subgroups, the GSEA 4.2 software was used to discover the Kyoto Encyclopedia of Genes and Genomes (KEGG) pathways notably enriched in the two risk groupings (the criterion was defined as FDR < 0.05).

### Correlation analysis of immunologic characteristics and m7G methylation

Immune infiltration by different algorithms was downloaded from TIMER2.0 (http://timer.cistrome.org/) and merged with the risk score. Then, the association between the risk model and immune infiltration was estimated derived from Spearman's rank correlation coefficient. The two risk subgroups were also compared using a single-sample gene set enrichment analysis (ssGSEA) to assess whether or not there was a massive distinction in immune function and immune cell enrichment. Moreover, the high- and low-risk group were compared in relation to the tumor microenvironment (TME) score, and expression of immune checkpoint-related genes. To explore the relationship between necroptosis-related lncRNAs and m7G methylation, we compared the expression of m7g-related genes between high- and low-risk group segmented according to this risk signature.

### Estimation of anticancer drug sensitivity for SKCM

The “pRRophetic” R package, founded by Genomics of Drug Sensitivity in Cancer (GDSC)(https://www.cancerrxgene.org/), was employed to forecast the difference in drug sensitivity for both risk groups.

### Consensus cluster analysis based on the hub lncRNAs

The ConsensuClusterPlus R package was implemented to separate SKCM patients into potential subgroups depending on the expression of hub lncRNAs by the k-means clustering algorithm. We set the maximum k value as 9 and determine the optimal K value through the combination of CDF and consensus matrices. Through using Rtsne and survival R package, we examined the subtype distribution and assessed the classification effectiveness including using t-distributed stochastic neighbor embedding (t-SNE), principal component analysis (PCA), and Kaplan–Meier survival analysis. Moreover, the immune status, m7G methylation-related genes, and clinical potential chemotherapeutic drug clusters were also analyzed simultaneously.

## Results

### Identify necroptosis-related lncRNAs in SKCM

**Figure 1:**
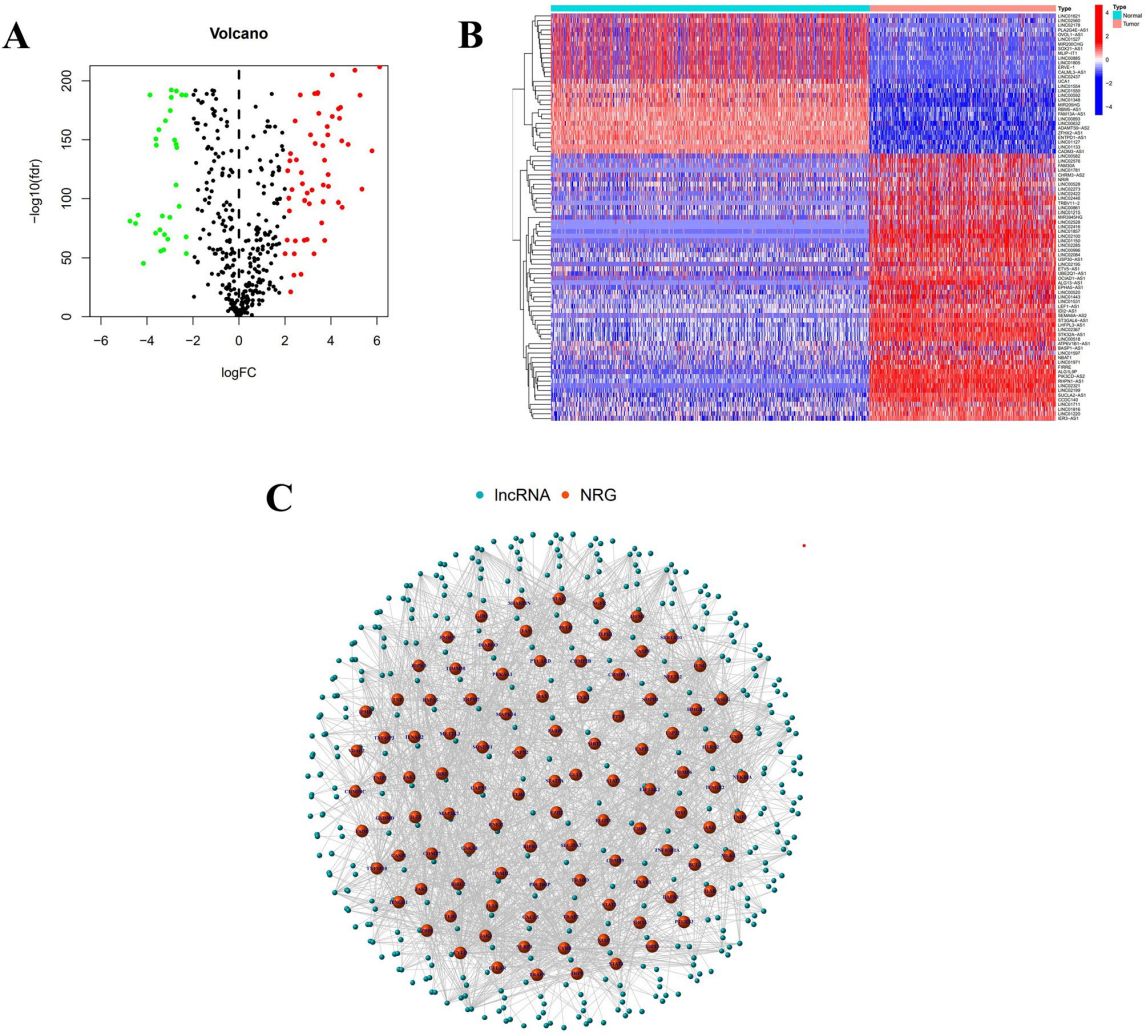
Identify necroptosis-related lncRNAs in SKCM. (**A**) Volcano diagram. (**B**) Heatmap of 87 lncRNAs' expression. (**C**) Network map of the link between lncRNAs and genes.

There are 386 necroptosis-related lncRNAs identified from the data of TCGA and GTEx, as the standard is the coefficients > 0.4 and *P* < 0.001. After that, flowing the differential expression analysis, 87 necroptosis-related lncRNAs were found to display significantly differential expression with the screen value as |logFC |> 2 and FDR < 0.05. Among the expression profile of these lncRNAs in tumor samples was 57 upregulated and 30 downregulated. The network map, heat map, and volcano map are shown in Fig. 1.

### Construction and verification of prognosis risk model

**Figure 2:**
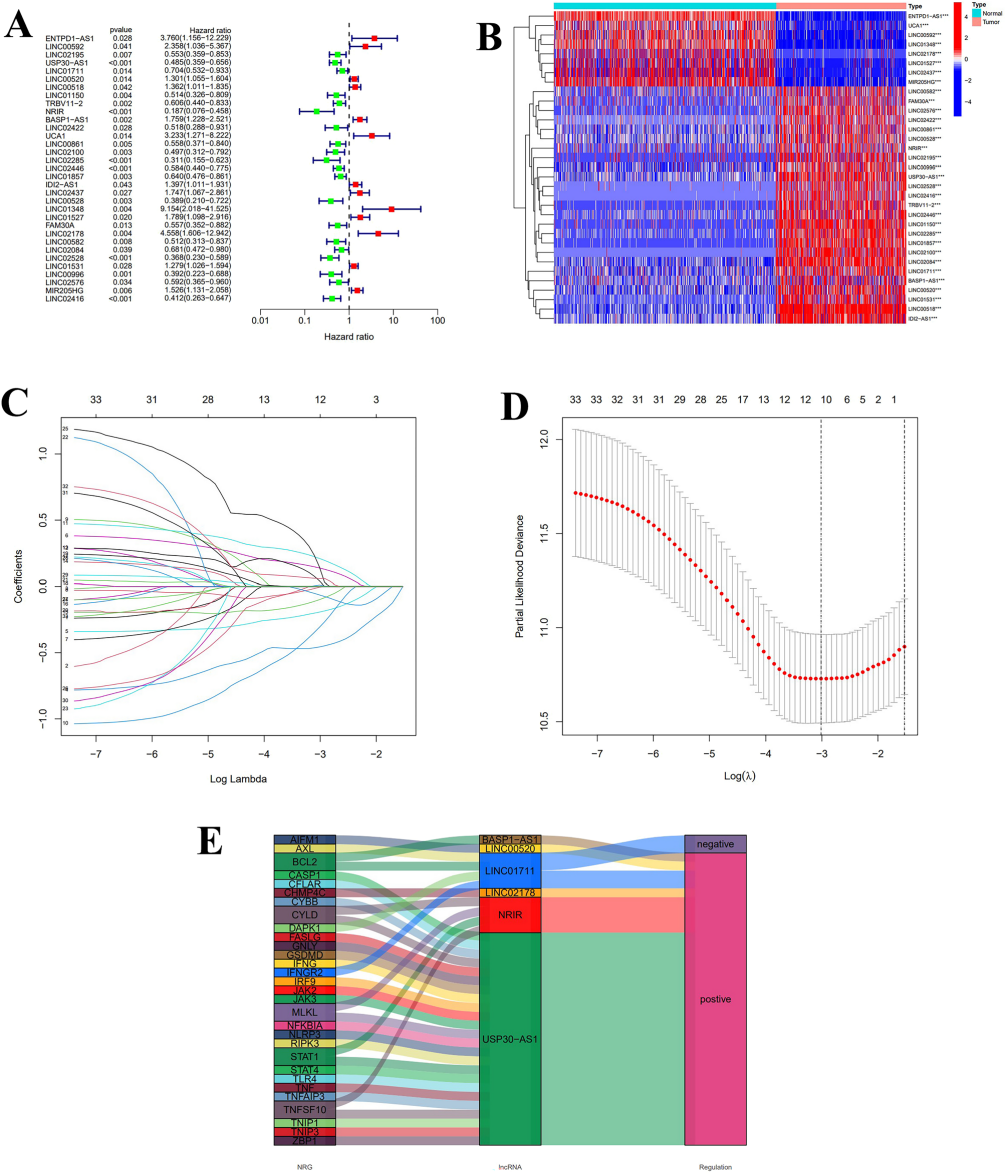
Screen and validate the necroptosis-related hub lncRNAs in SKCM. (**A**) 33 prognostic lncRNs in SKCM patients. (**B**) Expression of 33 prognostic lncRNAs in samples. (**C**) The LASSO model's tenfold cross verification results. (**D**) The optimal LASSO coefficients for risk model construction. (**E**) The relationship between 6 hub lncRNAs with necroptosis genes.

**Figure 3:**
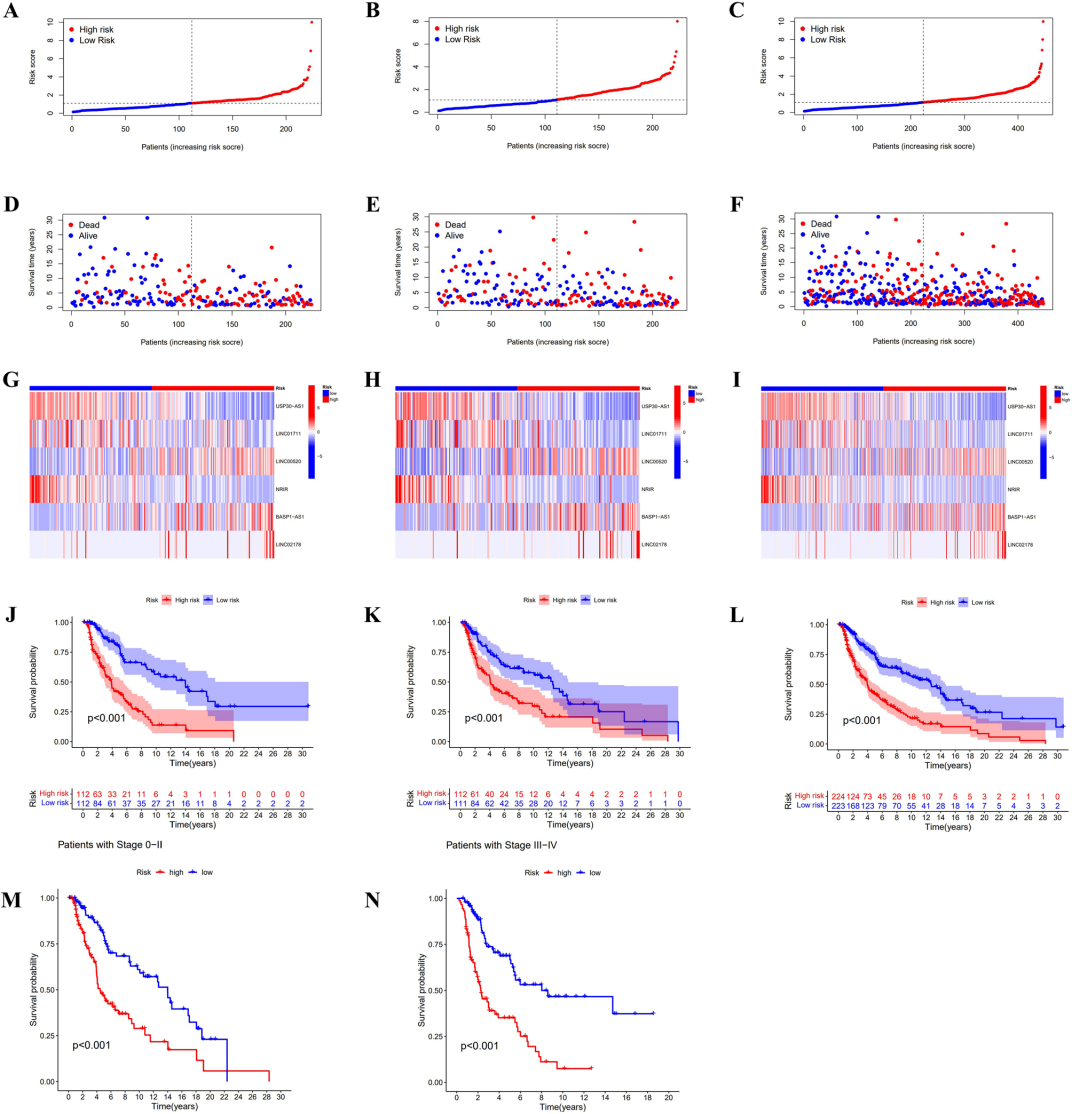
Forecasting capacity of the risk model. (**A**–**C**) Risk classification of SKCM patients in the training, testing, and overall set. (**D**–**F**) Relationship between survival condition and risk score in the training, testing, and overall set. (**G**–**I**) Different expressions of 6 hub lncRNAs in distinct risk scores of the training, testing, and overall set. (**J**–**L**) K–M curves' results of SKCM patients in the training, testing, and overall set. (**M**–**N**) K–M curves' results of SKCM patients in different cancer stages.

On the basis of uni-cox regression analysis, 33 potential necroptosis-related lncRNAs that are substantially linked with overall survival were reported (*p* < 0.05) (Fig. 2A). we also produced a heat map which shows how these lncRNAs were conveyed in both cancer and normal samples (Fig. 2B). Then, to construct the risk model, 6 hub necroptosis-associated lncRNAs were selected through LASSO regression analysis. Furthermore, we visualized the results of Lasso regression and cross-validation (Fig. 2C,D). Among these hub lncRNAs, only the relationship between LINC01711 with BCL2 and AXL was negative, while the rest were positive (Fig. 2E). The risk score was calculated by formula: USP30-AS1 × (− 0.6073) + LINC01711 × (− 0.3162) + LINC00520 × (0.2742) + NRIR × (− 0.6839) + BASP1-AS1 × (0.3524) + LINC02178 × (1.0015). The results of scatter plots and risk curves, which were consistent across the training, testing, and overall set, show that patients have shorter survival times as risk scores increase (Fig. 3A–F). USP30-AS1, LINC01711, and NRIR were clearly downregulated in the high-risk group, while LINC00520, BASP1-AS1, and LINC02178 presented opposite results in the heatmaps (Fig. 3G–I). The K–M curves were consistent across the training, test, and total pools, showing that patients with low-risk scores had significantly better overall survival (OS) than the high-risk group, implying a worse prognosis for high-risk patients (Fig. 3J–L). Furthermore, the K–M curves' results among the tumor's different stages were consistent with the front results (Fig. 3M,N).

### Construction of nomogram

**Figure 4:**
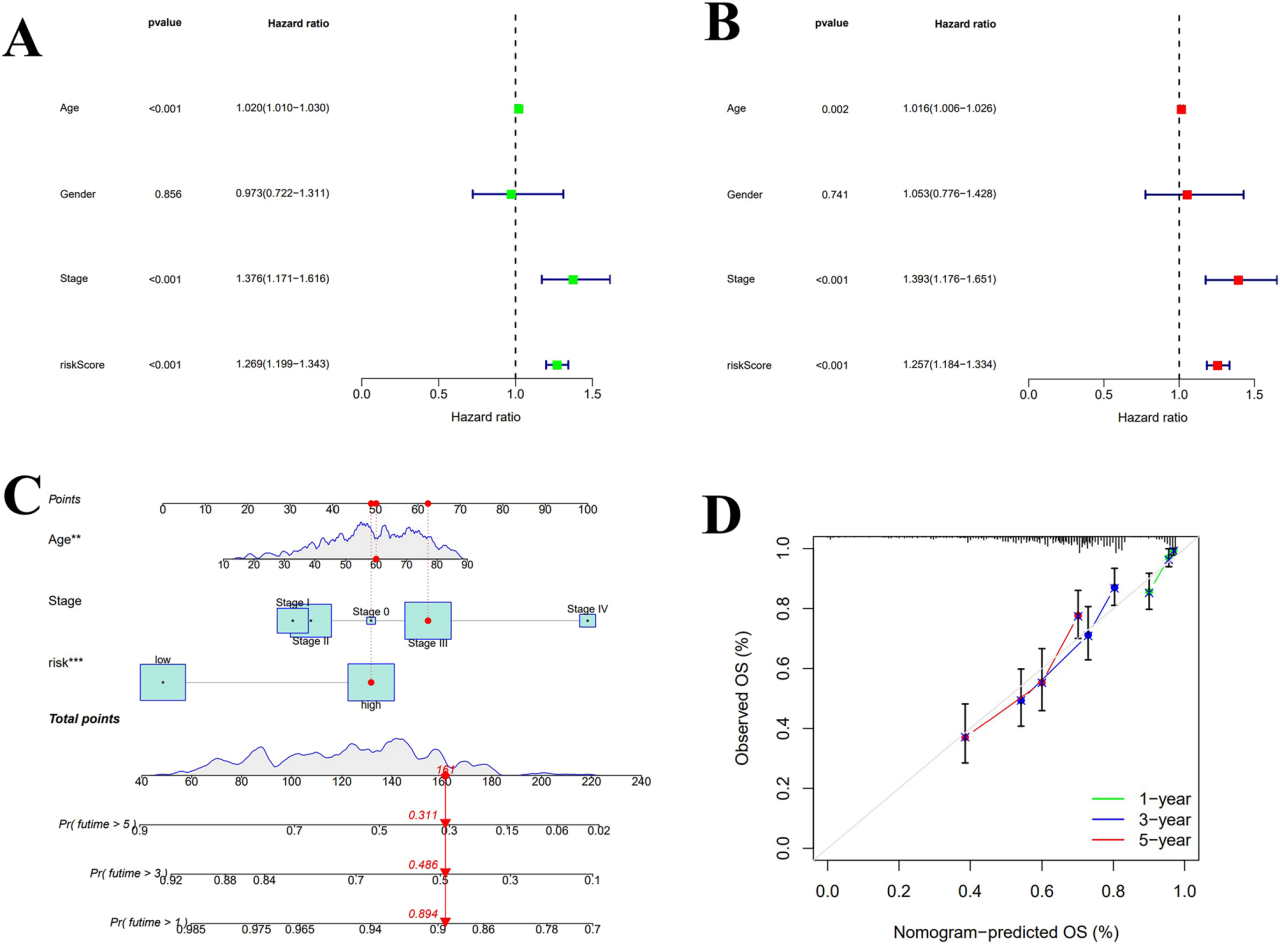
Nomogram and evaluation of the risk model. (**A**, **B**) Uni- and multi-Cox analysis of clinical variables and the risk score for OS. (**C**) Nomogram diagram of the risk model. (**D**) Calibration curves for 1-, 3-, and 5-year OS.

**Figure 5:**
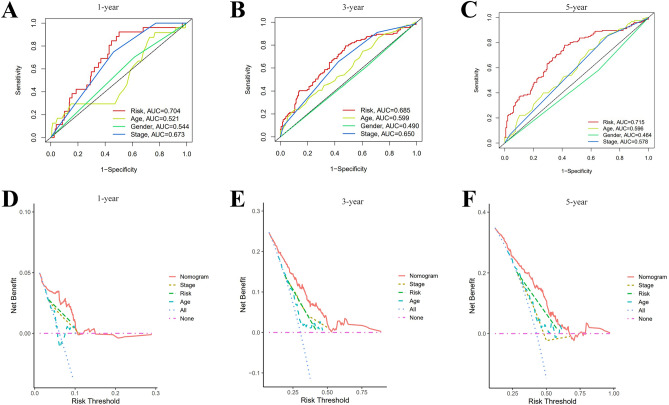
Verification of risk score as an independent predictor. (**A**–**C**) ROC curves for Nomogram at 1, 3 and 5 years compared to age, cancer stage and gender. (**D**–**F**) DCA curves for Nomogram at 1-, 3- and 5-year compared with age, cancer stage, and risk.

Compared with age, gender, and cancer stage, the hazard ratio (HR) of the risk score in uni-Cox and multi-Cox regression were 1.269 and 1.257 (*p* < 0.05) (Fig. 4A,B). Therefore, this risk signature may act as stable and specific biomarkers to predict the OS of melanoma patients. Upon the basis of risk score, age, and cancer stage, a nomogram was constructed to estimating the 1-, 3- and 5-year OS occurrences of SKCM patients (all *p* < 0.05 in multi-Cox) (Fig. 4C). Furthermore, the calibration curve result shows that the nomogram with an excellent concordance for 1-, 3- and 5-year (Fig. 4D). Additionally, by comparing the ROC curves of risk signature with age, gender, and cancer grade at 1-, 3- and 5-year, where the risk scores at 1-,3- and 5-year with AUC values of 0.704, 0.685 and 0.715 respectively, were significantly better than the other three clinical characteristics (Fig. 5A–C). We also constructed DCA curves for the nomogram at 1-, 3-, 5-year compared to age and cancer grade, all of which showed that the risk profile had a more accurate predictive power (Fig. 5D–F). These findings suggest that the Nomogram derived from this risk signature can accurately predict the prognosis of melanoma patients over the next 5 years.

### GSEA

**Figure 6:**
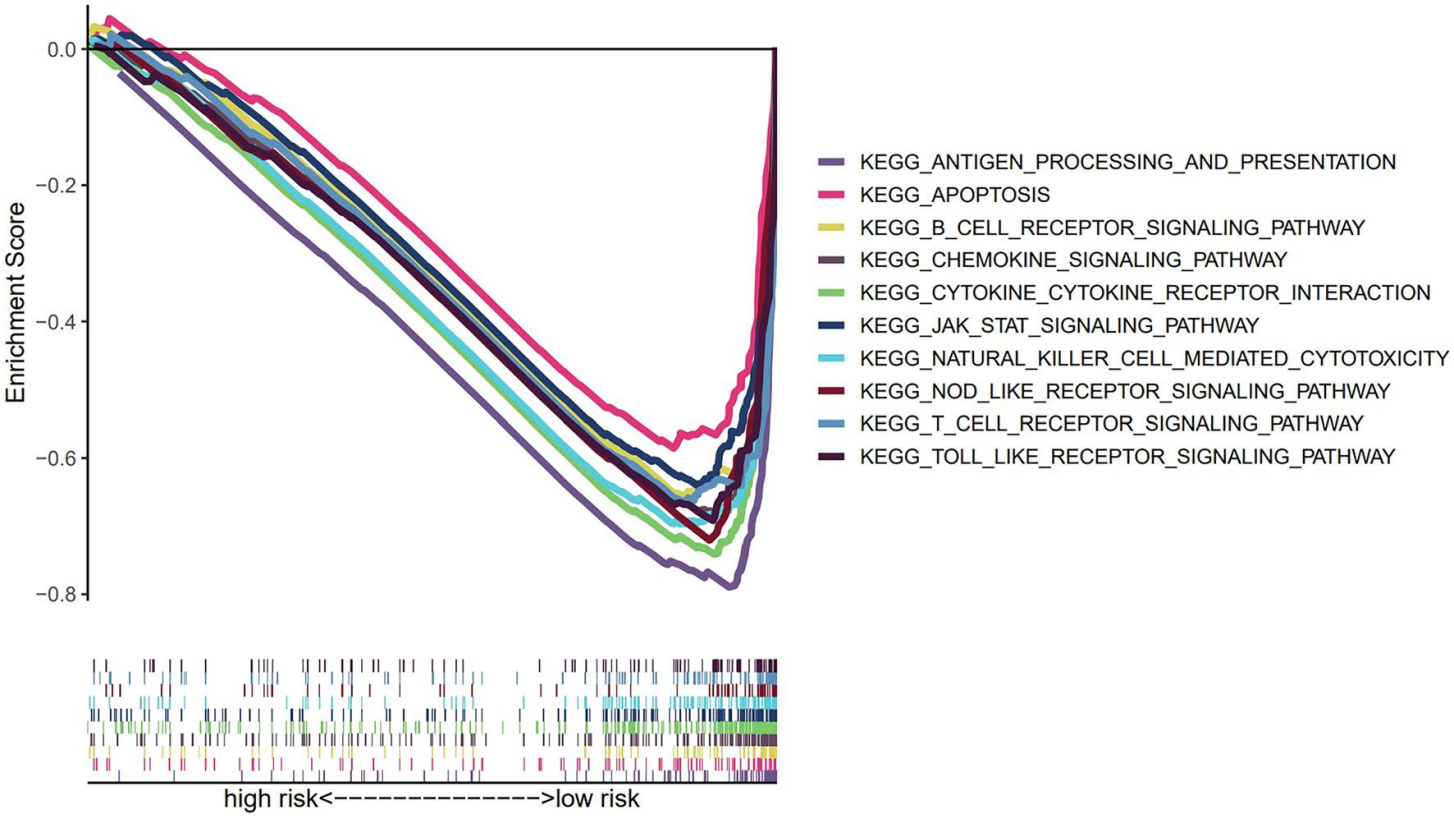
Richness of KEGG pathway via GSEA analysis.

The KEGG pathway enrichment analysis was carried out in the overall set to analyze the biological function of the risk signature. The top 10 pathways increased in the low-risk group were mainly associated with immunity function, including those involved in B cell, T cell, Toll-like receptor signaling, and natural killer cell-mediated cytotoxicity. Besides, the rest of the pathways were concerned with apoptosis, Cytokine-cytokine receptor interaction, and inflammation-induced immune response. Incredibly, there was no significant pathways enrichment in high-risk groups (FDR < 0.05) (Fig. 6).

### Investigation of immune factors and m7G methylation-associated genes in risk models

**Figure 7:**
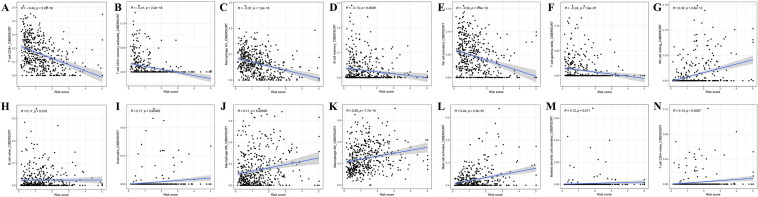
Potential links between distinct risk score and immune factors, immune checkpoint genes, and m7G genes. (**A**) Bubble diagram of immune cell relevance for various prediction platforms. (**B**–**D**) Boxplot for stromal, immune, and ESTIMATE scores. (**E**, **F**) Differentially expressed immune cells and immune function. (**G**, **H**) Differentially expressed immune checkpoint genes and m7G methylation genes.

**Figure 8:**
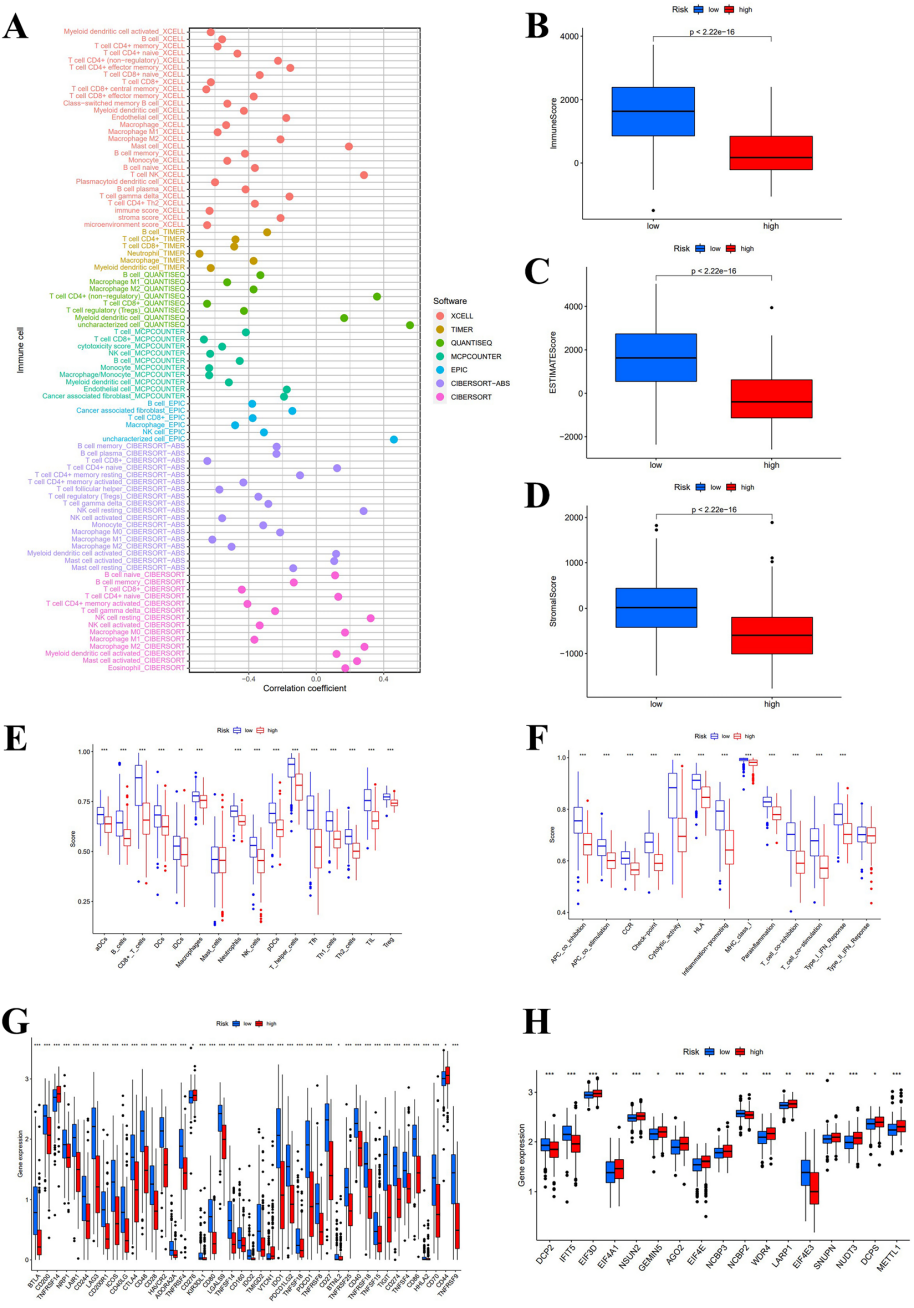
Prediction of immune cell infiltration. (**A**–**N**) Association between risk scores and immune infiltration based on the CIBERSORT software.

From the results on the bubble plot, the vast majority of immune cells were correlated with the low-risk group. The high-risk group was associated with a few immune cells, such as T cell NK and Mast cell at XCELL, Myeloid dendritic cell at QUANTISEQ, and Macrophage M1, M2 at CIBERSORT (Fig. 7A). For CIBERSORT platform, immune cell infiltration prediction showed that T cell CD8+, T cell CD4+ memory activated, Macrophage M1, NK cell activated, T cell gamma delta, and B cell memory was negative with risk scores. B cell naive, Myeloid dendritic cell activated, T cell CD4+ naive, Mast cell activated, Eosinophil, Macrophage M0, Macrophage M2, and NK cell resting were positive with risk scores (*P* < 0.05) (Fig. 8). The TME scores (containing stromal, immune, and ESTIMATE scores) were substantially higher in the low-risk group (*P* < 0.05) (Fig. 7B–D). We further observe that compared to the high-risk group, the low-risk group demonstrated significantly elevated levels of 15 immune cells score and 12 immune functions (***P < 0.001, **P < 0.01) (Fig. 7E,F). Only the scores of mast cell and type II IFN responses did not differ in the two groups. Above of this suggests that the low-risk group appears to be more strongly associated with the immune microenvironment. Furthermore, by differential expression analysis, the two groups showed significant differences in the expression profiles of 44 immune-related checkpoint genes (***P < 0.001, *P < 0.05) (Fig. 7G). Among them, TNFRSF14, CD276, VTCN1, and CD44 were enriched in the high-risk group, whereas the rest genes were concentrated in the low-risk group. Therefore, we can use risk grouping to rationalize the selection of immune checkpoint inhibitors. Between risk groups, there were statistically significant changes in the expression levels of 17 genes involved in m7G methylation. In comparison to the low-risk subgroup, the high-risk subgroup exhibited markedly lower amounts of DCP2, IFIT5, NCBP2, and EIF4E3, while higher levels of EIF3D, EIF4A1, NSUN2, GEMIN5, AGO2, EIF4E, NCBP3, WDR4, LARP1, SNUPN, NUDT3, DCPS, and METTL1 (***P < 0.001, **P < 0.01, *P < 0.05) (Fig. 7H).

### Relevance between risk model and drug sensitivity

**Figure 9:**
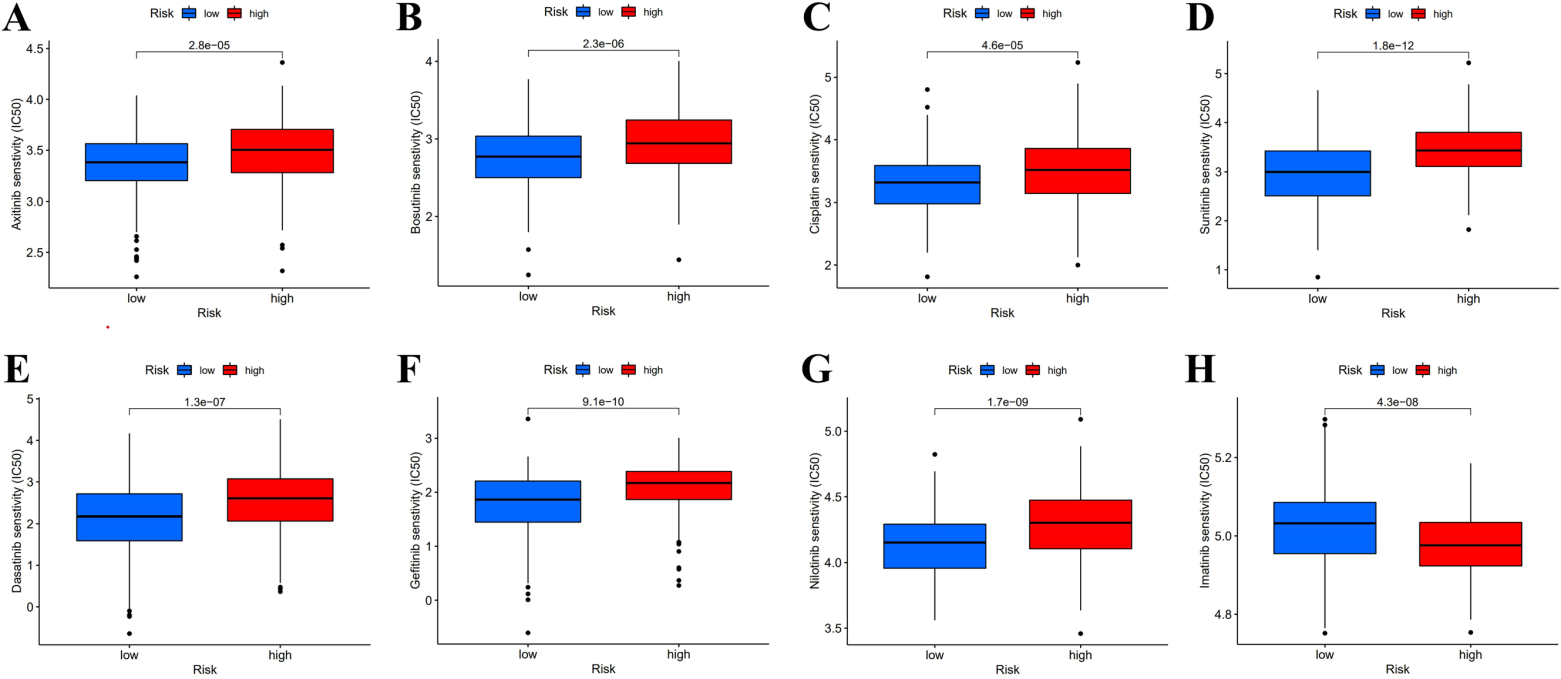
Drug prediction for SKCM patients. (**A**–**H**) IC50 prediction for Axitinib, Bosutinib, Cisplatin, Dasatinib, Gefitinib, Nilotinib, Sunitinib, and Imatinib at distinct risk groups.

Necroptosis-related hub lncRNAs correlate with several common melanoma therapeutic agents. Low-risk patients were more responsive to Axitinib, Bosutinib, Cisplatin, Dasatinib, Gefitinib, Nilotinib, and Sunitinib than than high-risk patients. In contrast, the finding for imatinib was polar opposite (*P* < 0.05) (Fig. 9). Therefore, individualized selection of sensitive drugs for the risk subgroup was expected to improve the effectiveness of clinical treatment.

### Differentiate between cold and hot tumors

**Figure 10:**
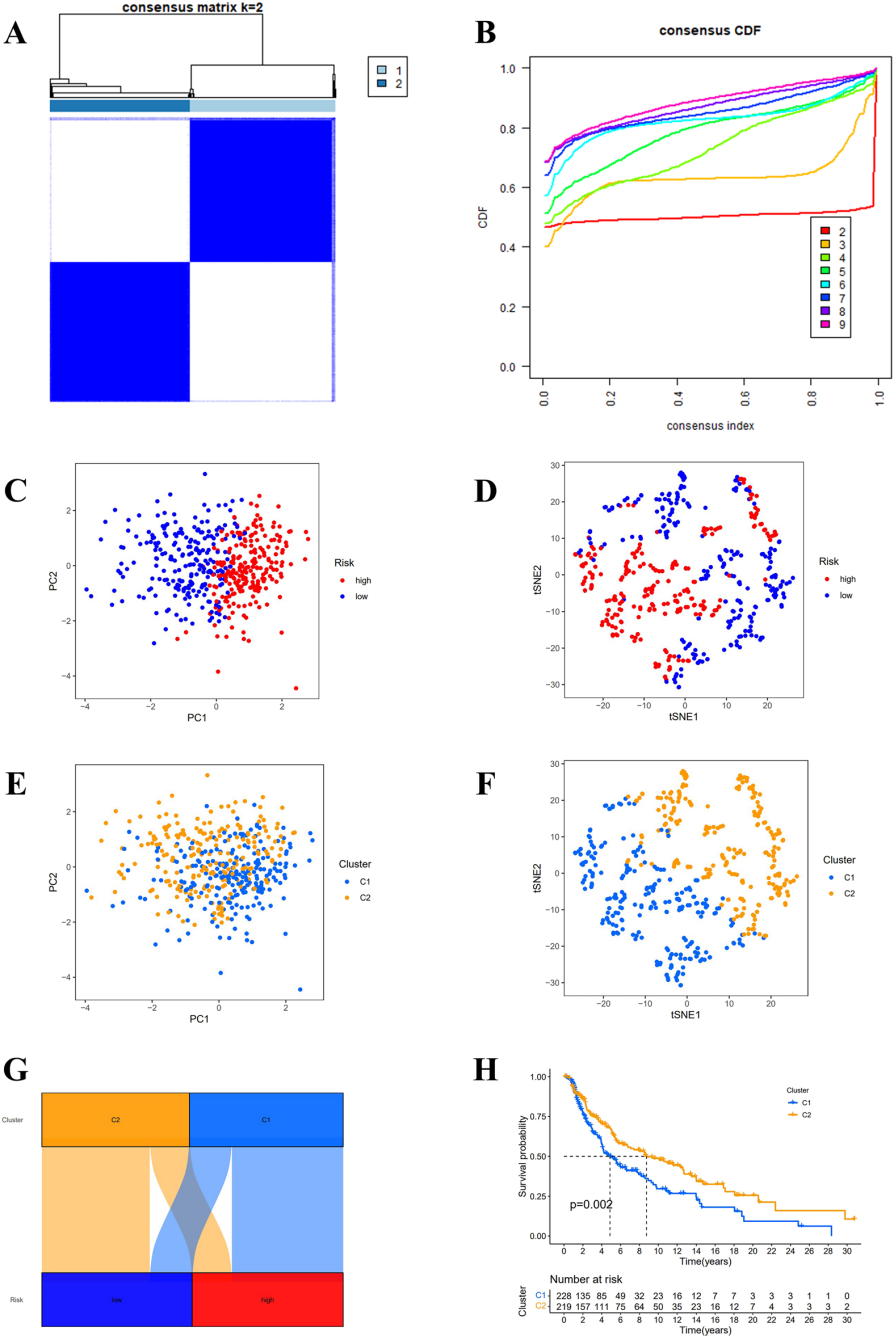
Distinguish between cold and hot tumors. (**A**, **B**) The optimal CDF diagram and consensus matrix plots of subgroups. (**C**–**F**) PCA and t-SNE diagram for risk groups and clusters. (**G**) Composition relationship between risk groups and clusters. (**H**) K-M curves' results of SKCM patients in clusters.

**Figure 11:**
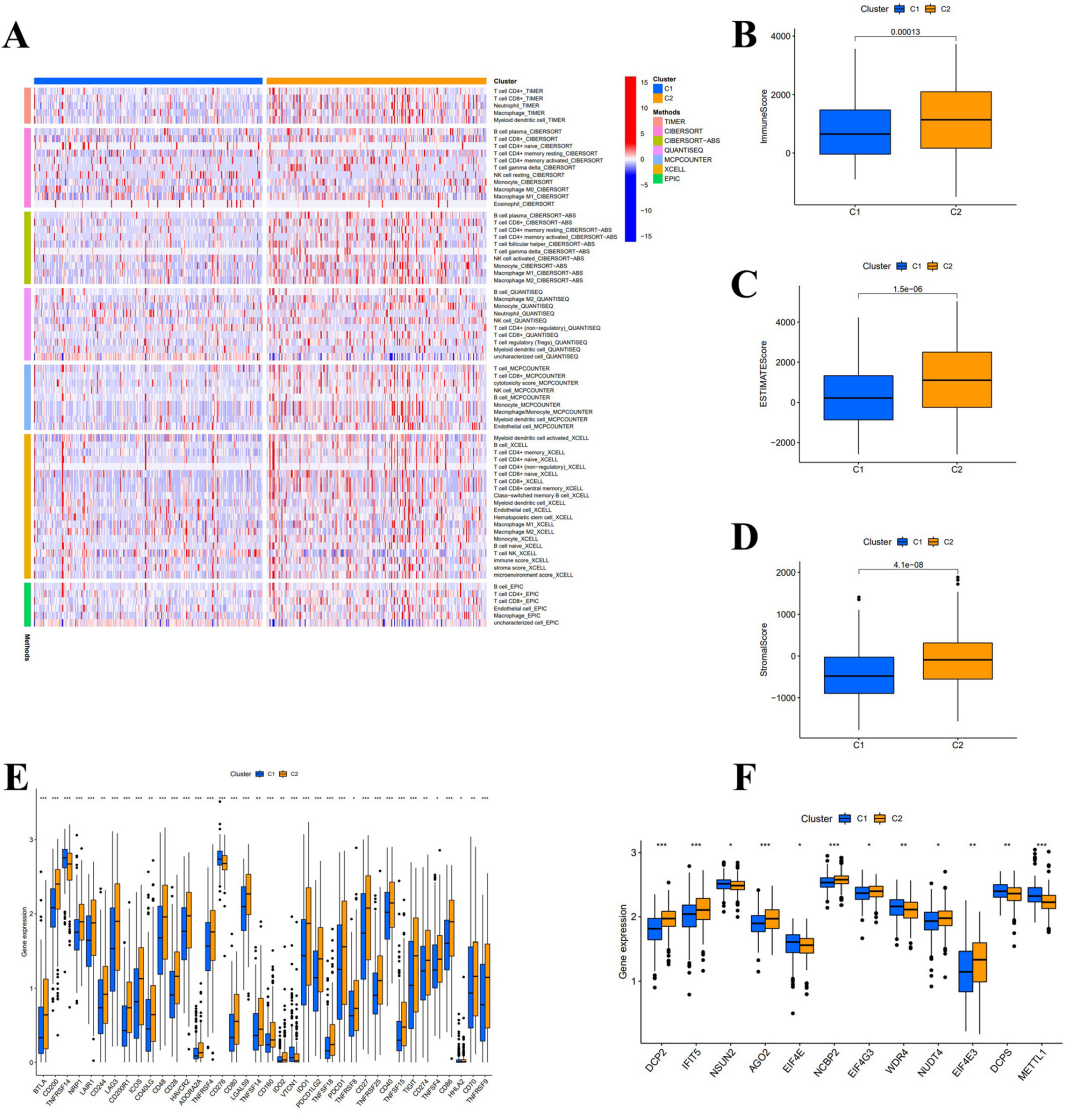
Potential correlation of immune factors, immune checkpoint genes, and m7G genes with hot and cold tumors. (**A**) Heatmap of immune cells for clusters. (**B**–**D**) Boxplot for stromal, immune, and ESTIMATE scores between clusters 1 and 2. (**E**, **F**) Differentially expressed immune checkpoint genes and m7G methylation genes between clusters 1 and 2.

**Figure 12:**
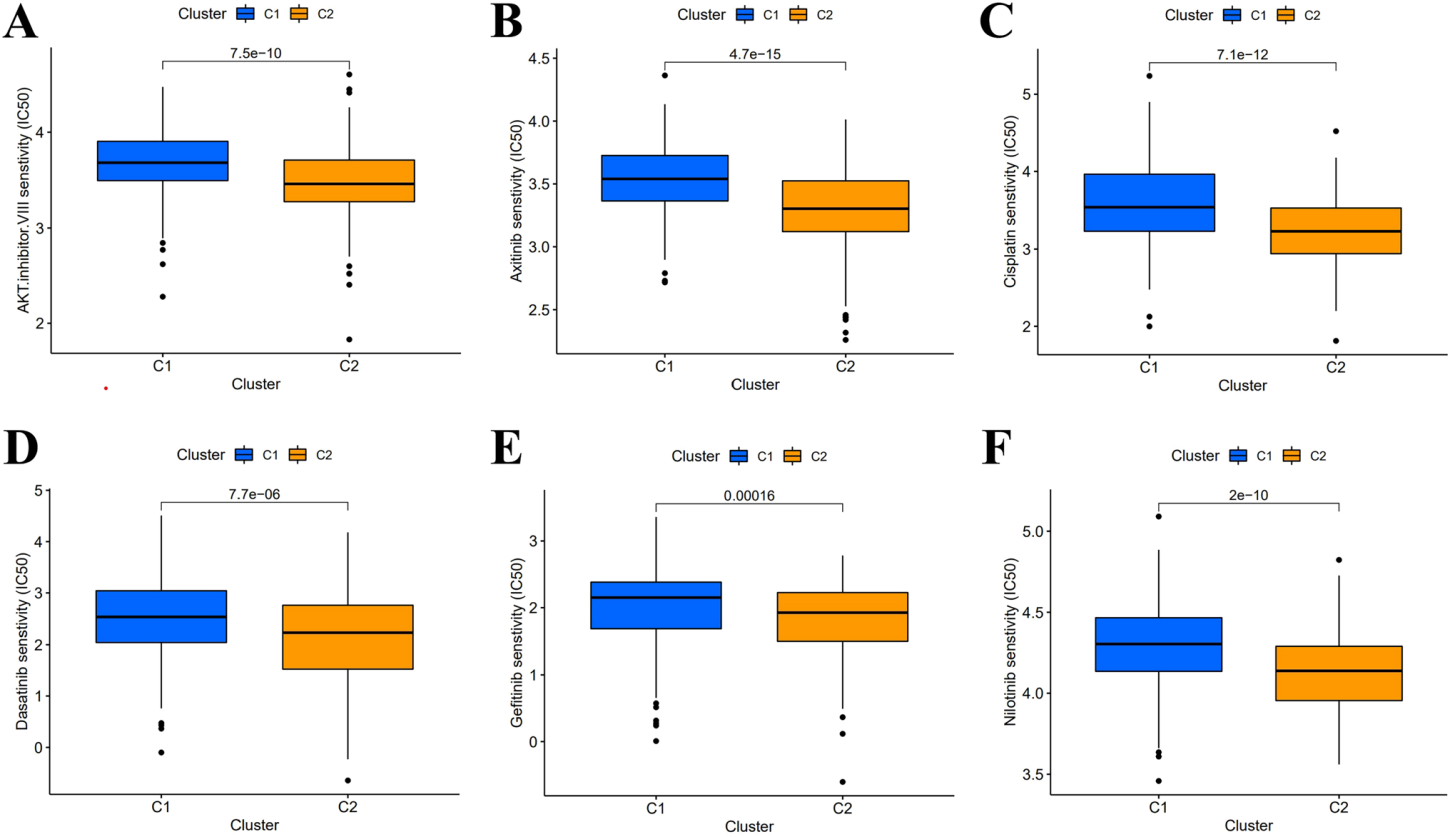
Drug prediction for hot and cold tumors. (**A**–**F**) IC50 prediction for cluster 1 and cluster 2 in AKT.inhibitor.VIII, Axitinib, Cisplatin, Dasatinib, Gefitinib, and Nilotinib.

According to the results shown in the CDF diagram, consensus matrix plots, and fragment plots, the optimal K value was 2 (Fig. 10A,B). Then, we reclassified SKCM patients into two clusters depending on the expression of hub lncRNAs. The results of PCA and t-SNE show that the distribution of samples in clusters does not exactly match the risk subgroups (Fig. 10C–F). The Sankey plots show the relationship between the two grouping methods, where Cluster1 strongly linked to the high-risk group and Cluster 2 to the low-risk group (Fig. 10G). Following the K–M analysis, Cluster 2 exhibited a superior survival outcome (*P* = 0.002) (Fig. 10H). By evaluating the immune microenvironment for clusters, we found that Cluster 2 had a higher degree of immune infiltration and a significantly higher TME score than Cluster 1 (*P* < 0.05) (Fig. 11A–D). Only TNFRSF14, CD276, and VTCN1 were significantly expressed in Cluster 1. Conversely, most immune checkpoint genes were significantly activated in Cluster 2 (***P < 0.001; **P < 0.01; *P < 0.05) (Fig. 11E), especially the HAVCR2, LAG3, and CD274 genes, which were thought to contribute to important functions in hot tumors. Accordingly, cluster 2 was considered the hot tumor. The expression levels of m7G methylation-related genes DCP2, IFIT5, AGO2, NCBP2, EIF4G3, NUDT4, and EIF4E3 were higher, while NSUN2, EIF4E, WDR4, DCPS, and METTL1 were lower in cluster 2 in comparison with cluster 1 (Fig. 11F). We also analyzed the differences in drug IC50 between clusters and showed that AKT.inhibitor.VIII, Axitinib, Cisplatin, Dasatinib, Gefitinib, and Nilotinib had a higher score in cluster 1 than in cluster 2 (*P* < 0.05) (Fig. 12). As a result, the above-mentioned 6 drugs were more effective against cluster 2, the hot tumor. These drugs may be used as an adjunctive treatment option to reverse the poor outcome of immunotherapy resistance.

## Discussion

For melanoma, the most lethal skin cancer, benefits for patients have vastly improved since the introduction of targeted treatments and immunotherapy^28–30^. However, the problems of inadequate response and drug resistance to BRAF/MEK and CTLA-4/PD-1 inhibitors are becoming increasingly tricky^31,32^. Necroptosis, as a form of immunogenic cell death, can overcome apoptosis resistance and effectively facilitate anti-cancer immunity^33,34^. Some lncRNAs have been found to have tumor-suppressor functions, which can eliminate adverse effects of immunotherapy resistance for cancer. On the other hand, some lncRNAs were known to help induce immune system evasion for cancer cells^24,25^. Therefore, the based targets of necroptosis-related lncRNAs would assist with immunotherapy in melanoma as a potential strategy to evade therapy failure.

In the present study, USP30-AS1, LINC01711, LINC00520, NRIR, BASP1-AS1, and LINC02178 were selected as the final components of the risk model. The Sankey diagram shows that only LINC01711 was negatively correlated with BCL2 and AXL among the hub lncRNAs. Silencing of BCL2 in melanoma cells inhibits the polarization of M2 macrophages, promoting anti-cancer immunity and interfering with tumor progression^35^. AXL was prominently expressed in the sentinel lymph nodes of melanoma, suggesting a correlation with metastasis^36^. Overexpression of both genes would affect the outcome of anti-PD-1 immunotherapy^37,38^. LINC02178 has been shown to be associated with lung adenocarcinoma and endometrial cancer, which may serve as a specific prognostic marker for cancer^39,40^. NRIR, firstly found to be associated with melanoma by our study, was positively correlated with CYLD, MLKL, STAT1, and TNFSF10. These genes interfere with melanoma cell progression, epigenetic alterations, the microenvironment of tumor cells, and the effect on immunotherapy^16,41,42^. Besides above 3 lncRNAs, the remaining hub lncRNAs have been linked to the development of melanoma. Existing studies suggest that USP30-AS1 and BASP1-AS1 may be interfere in melanoma cancer cell autophagy^43,44^. Multiple researches have demonstrated that cancer cells with high expression levels of RIPK3 and MLKL are more likely to undergo necroptosis and trigger immune responses that fight tumors^11,45^. USP30-AS1 was highly correlated with RIPK3 and MLKL in the present investigation, however the risk score decreased with increasing USP30-AS1 expression. However, the specific regulatory pathways of USP30-AS1 interfering with RIPK3 and MLKL in melanoma still need further investigation. LINC00520 may interfere with melanoma progression through the miR125b-5p/EIF5A2 axis^46^. LINC00520 expression was positively correlated with AIFM1, and high-risk scores, suggesting a poor outcome. However, the relevance of AIFM1 in melanoma remains uncertain. These obtained hub lncRNAs may provide a foundation for research addressing melanoma treatment failure.

Immune checkpoint inhibitors have transformed the treatment of patients with advanced melanoma, providing many patients with the opportunity to extend their survival^47^. However, many patients do not respond to treatment, and others experience varying degrees of adverse drug effects. Therefore, differentiating between cool and hot tumors by evaluating the immune microenvironment helps better direct immunotherapy interventions^48^. Hot tumors tend to have a high degree of immune infiltration and are enriched in many immune functions^49^. Necroptosis, a potentially novel mechanism of immune cell death, releases large amounts of immunogenic DAMPs. Stimulation of DAMPs may alter the immune resistance of the tumor, transforming it from a cold to a hot tumor. For these reason, necroptosis activation in cancer cells may boost immunotherapy's effectiveness^34^.

RNA methylation, a type of RNA modification, happens in both mRNA and lncRNA. Although m7G-related tumor research is still in its infancy, current studies indicate that it interfered with cancer development and can regulate the immune environment of cancer cells by regulating the expression of genes^50,51^. N7-methylguanosine (m7G) has been associated with modifications of a range of genes, particularly METTL1 and WDR4^52^. Our results show that the typical m7G methylation-related genes, METTL1, and WDR4, are more highly expressed in high-risk and cold tumors compared to the other two subgroups. This fantastic result suggests that inhibiting m7G methylation modifications may promote necroptosis in cancer cells and stimulate anti-tumor immunity. However, the concrete mechanisms of these relationships are unclear, which will facilitate future studies of m7G methylation of RNA in melanoma.

With the developing knowledge of the biology underlying melanoma, the combination therapeutic strategy for targeted genetic inhibitors with immune checkpoint inhibitors can significantly benefit advanced patients^29,31^. However, the rising incidence of drug resistance and complications has led to treatment failure and increased patient burden. In the present study, the sensitivity of some common adjuvant melanoma drugs not only differed between high- and low-risk groups but also hot and cold tumors. Therefore, drugs with significant sensitivity may modify the immune microenvironment of the tumor and reduce the adverse effects of immune escape as a complementary option in order to maximize the efficacy of immunotherapy and the survival time for metastatic melanoma patients as much as possible.

Although we attempted to construct the model by increasing the number of random groupings and cross-validation to make the risk model more accurate, there are still limitations. Above all, as a retrospective study, despite the relatively large sample size and the internal validation, external validation of other databases is still lacking. The reason is related to other datasets with smaller samples, and less complete information is less suitable for validation. Second, there is a dearth of experimental analysis to support the results of bioinformatics analyses. The predicted results for drug sensitivity are based on a pan-cancer public database. Hence, multicenter trial clinical efficacy data and prospective studies are required to demonstrate its viability. Future challenges include a deeper exploration of the mechanisms behind necroptosis-related lncRNAs in melanoma is needed.

## Conclusion

Our research successfully predicted the necroptosis-related hub lncRNAs in melanoma, and these lncRNAs provide novel potential targets to eliminate the adverse effects of immunotherapy resistance in melanoma. Furthermore, identifying hot and cold tumors and providing personalized treatment plans that improve the immune microenvironment would provide more significant benefits for advanced patients. Therefore, we consider that necroptosis-related hub lncRNAs have a significant role in aiding earlier detection and improving poor treatment outcomes in malignant melanoma.

## Data Availability

The data that support the findings of this study are available from the first author upon reasonable request. The datasets for analysis in this research were obtained from the open TCGA (https://portal.gdc.cancer.gov/) as well as the GTEX database (https://xenabrowser.net/).
